# Perceived causes and consequences of sexual changes after cancer for women and men: a mixed method study

**DOI:** 10.1186/s12885-015-1243-8

**Published:** 2015-04-11

**Authors:** Jane M Ussher, Janette Perz, Emilee Gilbert, The Australian Cancer and Sexuality Study Team

**Affiliations:** 1 Centre for Health Research, University of Western Sydney, Locked Bag 1797, Penrith South, 2751 Australia; 2 School of Social Sciences and Psychology, University of Western Sydney, Locked Bag 1797, Penrith South, 2751 Australia

**Keywords:** Cancer and sexuality, Sexual frequency, Sexual satisfaction, Relationships, Psychological distress

## Abstract

**Background:**

Previous research on cancer and sexuality has focused on physical aspects of sexual dysfunction, neglecting the subjective meaning and consequences of sexual changes. This has led to calls for research on cancer and sexuality to adopt an “integrative” approach, and to examine the ways in which individuals interpret sexual changes, and the subjective consequences of sexual changes.

**Method:**

This study examined the nature and subjective experience and consequences of changes to sexual well-being after cancer, using a combination of quantitative and qualitative analysis. Six hundred and fifty seven people with cancer (535 women, 122 men), across a range of reproductive and non-reproductive cancer types completed a survey and 44 (23 women, 21 men) took part in an in-depth interview.

**Results:**

Sexual frequency, sexual satisfaction and engagement in a range of penetrative and non-penetrative sexual activities were reported to have reduced after cancer, for both women and men, across reproductive and non-reproductive cancer types. Perceived causes of such changes were physical consequences of cancer treatment, psychological factors, body image concerns and relationship factors. Sex specific difficulties (vaginal dryness and erectile dysfunction) were the most commonly reported explanation for both women and men, followed by tiredness and feeling unattractive for women, and surgery and getting older for men. Psychological and relationship factors were also identified as consequence of changes to sexuality. This included disappointment at loss of sexual intimacy, frustration and anger, sadness, feelings of inadequacy and changes to sense of masculinity of femininity, as well as increased confidence and self-comfort; and relationship strain, relationship ending and difficulties forming a new relationship. Conversely, a number of participants reported increased confidence, re-prioritisation of sex, sexual re-negotiation, as well as a strengthened relationship, after cancer.

**Conclusion:**

The findings of this study confirm the importance of health professionals and support workers acknowledging sexual changes when providing health information and developing supportive interventions, across the whole spectrum of cancer care. Psychological interventions aimed at reducing distress and improving quality of life after cancer should include a component on sexual well-being, and sexual interventions should incorporate components on psychological and relational functioning.

## Background

### Changes to sexuality after cancer

With cancer survival rates at 5 years currently over 60% [1], increasing numbers of individuals are living with the disease, leading to a focus on the quality of life of survivors, and their families. Sexual well-being is a central component of quality of life [2], and there is a growing body of research demonstrating the association between cancer and changes to sexuality and intimacy, primarily resulting from the impact of cancer treatment [3]. These changes can lead to significant distress, which in some instances can be experienced as the most difficult aspect of life following cancer [4].

Research examining changes to sexuality after cancer has primarily focused on cancers that directly affect the sexual or reproductive body. In men, this has involved examination of sexual changes following prostate and testicular cancer treatment, which include erectile dysfunction [5,6], diminished genital size, weight gain, urinary incontinence [7,8], reductions in sexual desire and enjoyment, as well as negative body image [9-12]. Research on sexual changes for women with cancer has primarily focused on the impact of treatments for gynaecological or breast cancer, which include anatomical changes [13-15], tiredness [16], vaginal pain or dryness [17,18], as well as negative feelings of sexual un-attractiveness [19,20], and changes to sense of femininity [21,22]. This can result in reductions in sexual desire [23], and response [24,25], leading to decreased frequency of sex [26], and lack of sexual pleasure or satisfaction [27,28].

There is growing evidence that individuals with cancers that do not directly affect the sexual or reproductive body can also experience a reduction in sexual interest and sexual activity, changes to body image and feelings of sexual competency, as well as sexual dysfunction, and alterations to sexual self-esteem [29,30]. For example, researchers have reported sexual changes in people with lymphatic [31,32], colon [33], head and neck [34,35], colorectal [36-38], bladder [39], and lung cancers [40]. However, interventions to ameliorate the impact of sexual changes have largely focused on sexual or reproductive cancers [41], and health professionals have been reported to be less likely to discuss sexual changes with individuals or couples experiencing a non-reproductive cancer [42-44]. This suggests that the sexual needs and concerns of those experiencing a wide range of non-reproductive cancers may not be acknowledged or addressed. There is a need for further research examining the nature and subjective experience of changes to sexuality, for both women and men, across a range of cancer types. This is one of the aims of the present study.

### Subjective experience and consequences of sexual changes after cancer

Previous research on cancer and sexuality has focused on sexual functioning, or on examination of factors that predict sexual dysfunction, focusing on demographic variables [45-48], type of treatment [36,49,50], or relationship context [51]. Whilst this body of work is important in identifying factors that may be associated with sexual difficulties after cancer, little attention has been paid to the subjective and social meaning and consequences of such sexual changes [37,52]. This has led to calls for research on cancer and sexuality to adopt an “integrative” approach ([53], p.3717), recognising physical, psychological, and relational aspects of experience [37], as well as the ways in which social constructions of sex influence the experience of sexual change [52,54,55]. In this vein, there is a substantial body of research examining the psychological consequences of sexual changes experienced after cancer [6,30,36,37,48,56-58], suggesting that sexual difficulties are associated with lower quality of life, and higher levels of distress. There is also evidence that sexual changes after cancer can impact upon the couple relationship [59], due to emotional distance between couples [60], feeling unwanted by one’s partner [16], negative thoughts about sexual contact [61], or difficulty with couple communication [62,63].

Previous research on psychological and relational aspects of changes to sexuality after cancer has primarily used quantitative methods of data collection. Whilst this provides important information about the nature and psycho-social correlates of sexual changes, it does not enable analysis of the subjective experience and meaning of such changes for people with cancer [64]. There has been some qualitative research that has examined changes to sexuality after cancer see [11,16,21], and the ways in which socio-cultural discourses shape the experience and interpretation of sexuality [52,65]. However, this research has been based on a small number of participants, primarily with sexual or reproductive cancers, which limits insights into the experience of individuals with other types of cancer.

### Study aims and research questions

There is a need for a larger mixed method study across a range of relationship contexts and cancer types to examine the nature and subjective experience of changes to sexual well-being after cancer, as well as the perceived individual and relational consequences, using a broad definition of sexual activity. This is the aim of the present study. We are adopting an integrative material-discursive-intrapsychic (MDI) model [64,66], which conceptualises sex and sexual well-being as a multi-faceted construct [67], wherein the effects of cancer and its treatment result from the interconnection of material, discursive and intrapsychic factors. This includes the *materiality* of embodied sexual changes after cancer, including changes in desire and functioning, and anatomical changes resulting from cancer treatment, as well as the material context of people’s lives, such as whether they are in a relationship or have partner support; changes which occur at an *intrapsychic* level, such as reductions on psychological well-being, and changes to sexual self-schema [68], identity [69], or body image [61]; and socio-cultural representations and *discourses* which shape the experience and interpretation of sex, telling us what is ‘normal’ and ‘abnormal’ sexual behaviour [55]. In contrast to bio-psycho-social models of experience [70], which conceptualise biology, psychology and social factors as independent, the MDI model conceptualises material, intrapsychic and discursive factors as inseparable. For example, the experience of *material* changes to sexual functioning which result from prostate cancer treatment – erectile dysfunction and reductions in sexual desire - is inseparable from *intrapsychic* responses to such changes – feelings of loss of manhood and depression [5] – and the discursive context which positions erectile functioning as sign of masculinity, and performance of coital sex as ‘real sex’ [71].

Within this MDI framework, we addressed the following questions: What is the nature and subjective experience of sexual changes experienced after cancer, for women and men, across reproductive and non-reproductive cancers? What are the perceived causes and consequences of such changes, for the person with cancer, and for their intimate relationship?

## Method

### Participants

Six hundred and fifty seven people with cancer (535 women, 122 men) took part in the study, part of a larger mixed methods project examining the construction and experience of changes to sexuality after cancer [43,51,55,72]. The average age of survey participants was 52.6 years (range 19-87) and cancer was diagnosed on average five years prior to participation in the study (range 1 month – 40 years). The majority (95%) identified as from an Anglo-European-Australian background, with the remainder identifying as from Asian, Aboriginal and Indian subcontinent backgrounds. The following cancer types were reported: breast (64.7%), prostate (13.2%), gynaecological (6.8%), haematological (5.6%), gastrointestinal (2.3%), neurological (1.5%), skin (1.5%), head and neck (0.9%), respiratory (0.2%), and other (0.4%). There were no significant demographic differences between participants with sexual or reproductive cancers (breast, prostate, gynaecological), and non-reproductive cancers. Eighty-six per cent of participants were currently in a relationship, 77% living together, with the average relationship length being 20 years (range 2 months-53 years). Ninety five per cent of participants identified as heterosexual, the remainder self-identifying as gay men (1.9%), lesbian (3%), or as poly-sexual (0.1%). Sample characteristics are presented in Table 1.

**Table 1:** **Sample characteristics by gender**

	Women		Men		Test for group difference	Significance	Effect size
Variable	*n*	*M (SD)*	*n*	*M (SD)*	*F*	*p*	*η2*
Patient age	535	50.7 (10.9)	122	61.1 (14.3)	79.01	<0.001	0.108
Years since first diagnosis	533	4.9 (5.3)	122	5.3 (5.4)	0.53	0.468	0.001
Length of current relationship	515	19.8 (13.7)	118	25.7 (16.8)	16.55	<0.001	0.026
	*n*	%	*n*	%	χ^2^	*p*	*φ*
Cancer type:					519.19	<0.001	0.364
Breast	425	80	-	-			
Gynecologic	45	8.5	-	-			
Prostate	-	-	87	72.5			
Genitourinary (other)	4	0.8	7	5.8			
Hematological/Blood	23	4.3	14	11.7			
Digestive/Gastrointestinal	11	2.1	4	3.3			
Neurologic	6	1.1	4	3.3			
Skin	8	1.5	2	1.7			
Other^a^	9	1.7	2	1.7			
Cancer classification:					10.52	.001	0.127
Sexual cancer type	474	89.3	94	78.3			
Non-sexual cancer type	57	10.7	26	21.7			
Stage of disease:					27.19	<0.001	0.188
No longer detectable/In remission	430	80.8	71	58.7			
Receiving treatment	16	3.0	7	5.8			
Other^b^	86	16.2	43	35.5			
Relationship status:					3.12	0.374	0.032
Partnered – Living together	414	77.4	96	78.7			
Partnered – Not living together	34	6.4	10	8.2			
Not in a relationship	76	14.2	16	13.1			
Other/Not specified	11	2.1	-	-			
Sexual identity:					405.16	<0.001	0.858
Heterosexual	434	96.7	92	91.1			
Non Heterosexual	15	3.3	9	8.9			
Current sexual relationship:							
Yes	404	76.2	87	71.9			
No	126	23.8	34	18.6	0.99	0.319	0.039

Note ^a^ “Other” includes: Respiratory/Thoracic, Head & Neck, various, each less than 1%; ^b^ “Other” includes: a new different cancer; active monitoring; outcome not specified; η2 eta-squared; φ Phi coefficient.

We recruited participants nationally through cancer support groups, media stories in local press, advertisements in cancer specific newsletters, hospital clinics, and local cancer organisation websites and telephone helplines. Two individuals, a person with cancer and a partner, nominated by a cancer consumer organisation acted as consultants on the project, commenting on the design, method and interpretation of results. We received ethics approval from the University of Western Sydney Human Research Ethics Committee, and from three Health Authorities, from which participants were drawn.

### Measures

Participants completed an online or postal questionnaire examining their experiences of sexuality and intimacy post-cancer, using a combination of closed and open-ended survey items. The survey included standardised measures of sexual and relationship functioning, psychological well-being, and quality of life, reported elsewhere [51], as well as measures of sexual satisfaction, sexual frequency, changes in sexual activities, and perceived causes and consequences of sexual changes, reported in the present paper.

#### Sexual frequency

Participants were asked to report “how frequently did you engage in sexual activity (e.g. sexual intercourse, masturbation, oral sex)?” before the onset of cancer and currently, on a five point scale: never, rarely (less than once a month), sometimes (more than once a month, less than twice a week), often (more than twice a week) and every day. This item was drawn from the Changes in Sexual Functioning Questionnaire (CSFQ-14) [73], a validated instrument which evaluates sexual dysfunction, and modified to include the ‘before the onset’ of cancer ratings.

#### Cause of changes to sexual frequency

Participants who indicated that sexual frequency had changed, were asked to indicate what factors were perceived to be the cause of such change, using a yes/no response. These factors were: medication, surgery, general pain, loss of feeling, tiredness, sex specific difficulties (vaginal dryness, erectile difficulties), body changes, appearance changes, feeling unattractive, relationship change, psychological problems, stress, getting older, and other (self-nominated).

#### Change in sexual activities

A single item sexual measure developed as part of the study was used to assess “have your sexual activities changed since the onset of cancer?” using a yes/no response.

#### Nature of change in sexual activities

Participants were then asked “If yes, please indicate the types of sexual activities you engaged in now, and before the onset of cancer (please tick as many boxes as appropriate): kissing; petting, caressing and stroking; masturbating alone; masturbating with your partner; oral sex; sexual intercourse (vaginal and anal); use of sex toys; other (self-nominated)”.

#### Sexual satisfaction

A single item developed as part of the study was used to assess sexual satisfaction currently, and before cancer, using a 5 point Likert scale, ranging from could not be better, to could not be worse. The wording of the question was: “Below is a rating scale upon which we would like you to record your personal evaluation of how satisfying your sexual relationship is. The rating is simple. Make your evaluation by placing a tick in the appropriate box in each of the two columns that best describes your relationship as it is “Currently” and how it was “Before the Onset of Cancer””.

#### Open ended survey items

Participants provided qualitative responses to the following open ended questions: “What do you think are the causes of any changes in the type of sexual activities you engage in since the onset of cancer?”; “how have any changes to your sexuality since the onset of cancer made you feel about yourself and your relationship?”

#### In-depth interviews

At the completion of the survey, participants indicated whether they would like to be considered to take part in a one-to-one interview, to discuss changes to sexuality in more depth, as well as experiences of communication and information provision about sexuality from health professionals, the letter reported elsewhere [74]. Of the 657 survey respondents, 274 responded positively to the invitation. We purposively selected 44 people with cancer for interview (23 women, 21 men) representing a cross section of cancer types and stages, gender, and sexual orientation. The average age of interviewees was 54.6 years, with 50% experiencing a reproductive cancer (prostate, breast, gynaecological, anal) and 50% a non-reproductive cancer (colorectal, melanoma, lymphoma, leukaemia, kidney, bladder, and brain). Individual semi-structured interviews, lasting on average 60 minutes, were conducted by either a woman or man interviewer, on a face-to-face (7) or telephone basis (72). Participants were given a choice as to the mode of interview (telephone or face to face), and asked if they had a preference about the gender of the interviewer: the majority had no preference for gender, but chose telephone modality. Telephone interviews have previously been recommended for interviews regarding sensitive, potentially embarrassing topics [75], such as cancer and sexuality, and pilot interviews indicated that they were an effective modality to utilise in this study. Prior to the interview, participants were sent an information sheet and consent form to read and sign, as well as a list of the interview topics, including: changes to sexuality and intimacy; and experiences of communication and information provision about sexuality with health professionals. All of the interviews were transcribed verbatim.

### Analysis

#### Quantitative analysis of closed responses

The McNemar Chi-Square test for paired samples was used to test before cancer/after cancer differences within gender and cancer classification groups on ratings of sexual frequency and sexual satisfaction. To allow for dichotomous analysis and facilitate interpretation, ratings of sexual frequency were recoded into ‘never or rarely’ and ‘sometime, often and everyday’, whereas ratings of sexual satisfaction were recoded into ‘highly unsatisfying or unsatisfying’ and ‘adequate, satisfying and highly satisfying’ reflecting the direction and meaning of the original Likert scales. The McNemar Chi-Square test was also used to assess differences in frequency data for changes in sexual activities before and after cancer separately for women and men. The Fisher’s Exact Test (FET) was performed upon the categorical data associated with the perceived causes of changes in sexual frequency. In these analyses, the FET calculates the exact probability of significant differences in the reported assignments of women and men. An alpha level of .05 was used for all statistical tests, and 95% confidence intervals (CI) are reported for principal outcomes.

#### Qualitative analysis of open ended responses and interviews

The analysis was conducted using theoretical thematic analysis [76], using an inductive approach, with the development of themes being data driven, rather than based on pre-existing research on sexuality and cancer. In the analysis, our aim was to examine data at a latent level, examining the underlying ideas, constructions and discourses that shape or inform the semantic content of the data, interpreted within a material-discursive-intrapsychic theoretical framework [77]. All of the interviews were transcribed verbatim, and the answers to open ended questions collated. One of us read the resulting transcripts in conjunction with the audio recording, to check for errors in transcription. Detailed memo notes and potential analytical insights were also recorded during this process. A subset of the interviews and open ended questions was then independently read and reread by two of us to identify first order codes such as “embodied changes”, “emotional distress”, “relational issues”, “interactions with health professionals”, or “support needed”. The entire data set was then coded using NVivo, a computer package that facilitates organization of coded qualitative data. All of the coded data was then read through independently by two members of the research team. Codes were then grouped into higher order themes; a careful and recursive decision making process, which involved checking for emerging patterns, for variability and consistency, and making judgements about which codes were similar and dissimilar. The thematically coded data was then collated and reorganized through reading and rereading, allowing for a further refinement and review of themes, where a number of themes were collapsed into each other and a thematic map of the data was developed. In this final stage, a number of core themes were developed, which essentially linked many of the themes. These included the impact of sexual changes on self and identity [78], communication with health professionals [74], and renegotiation of sexuality [55], reported elsewhere, as well as perception of causes of sexual changes, emotional consequences of sexual changes, and impact of sexual changes on relationships, the focus of the present paper. Following analysis, the data were organised and presented using a conceptually clustered matrix [79], with exemplar quotes drawn from both the interviews and open ended survey questions provided in tables to illustrate each of the themes. The key to the quotes is: M/W = man or woman; age; gay/lesbian/heterosexual; cancer type.

## Results

### Subjective experience of changes to sexual frequency, sexual satisfaction and sexual activities after cancer

#### Sexual frequency

Table 2 presents the data on sexual frequency before and after cancer, for men and women, across both reproductive (breast, gynaecological and prostate) and non-reproductive cancers (all other cancers); age band (below and above age 55); years since diagnosis (less or more than two years); and current relationship duration (less or more than 15 years). There was a significant reduction in sexual frequency for both women (χ^2^ (1, 530) = 186.92, *p* < .001) and men (χ^2^ (1, 122) = 27.23, *p* < .001), with 11.9% of women and 13.1% of men reporting that sex occurred never or rarely before cancer, compared to 52.5% of women and 41% of men making this report after cancer. This pattern of a reported reduction in frequency occurred across all age, cancer type, years since diagnosis and relationship length categories: ≤55 years of age (χ^2^ (1, 389) = 130.05, *p* < .001) and ≥56 years of age (χ^2^ (1, 260) = 83.16, *p* < .001); reproductive (χ^2^ (1, 564) = 209.76, *p* < .001) and non-reproductive cancers (χ^2^ (1, 82) = 26.26, *p* < .01); ≤2 years since diagnosis (χ^2^ (1, 274) = 83.03, *p* < .001) and ≥3 years since diagnosis (χ^2^ (1, 375) = 130.06, *p* < .001); and ≤15 years in current relationship (χ^2^ (1, 262) = 69.77, *p* < .001) and ≥16 years in current relationship (χ^2^ (1, 366) = 141.74, *p* < .001).

**Table 2:** **Reports of sexual frequency before and after the onset of cancer by gender, age, cancer classification, time since diagnosis and relationship duration**

**Sexual frequency rating**	**Women %**			**Men %**		
** After Cancer**	Never or Rarely	Sometime; Often; Everyday	Total	Never or Rarely	Sometime; Often; Everyday	Total
**Before Cancer**						
Never or Rarely	76.2 (48)	23.8 (15)	11.9 (63)	81.2 (13)	18.8 (3)	13.1 (16)
Sometime; Often; Everyday	49.3 (230)	50.7 (237)	88.1 (467)	34.9 (37)	65.1 (69)	86.9 (106)
Total	52.5 (278)	47.5 (252)	(530)	41.0 (50)	59.0 (72)	(122)
	*χ*^2^ = 186.92***			*χ*^2^ = 27.23***		
	**≤55 Years of Age**			**≥56 Years of Age**		
** After Cancer**	Never or Rarely	Sometime; Often; Everyday	Total	Never or Rarely	Sometime; Often; Everyday	Total
**Before Cancer**						
Never or Rarely	68.3 (28)	31.7 (13)	10.5 (41)	86.8 (33)	13.2 (5)	14.6 (38)
Sometime; Often; Everyday	48.0 (167)	52.0 (181)	89.5 (348)	44.6 (99)	55.4 (123)	85.4 (222)
Total	50.1 (195)	49.9 (194)	(389)	50.8 (132)	49.2 (128)	(260)
	*χ*^2^ = 130.05***			*χ*^2^ = 83.16***		
	**Reproductive Cancer**			**Non-Reproductive Cancer**		
** After Cancer**	Never or Rarely	Sometime; Often; Everyday	Total	Never or Rarely	Sometime; Often; Everyday	Total
**Before Cancer**						
Never or Rarely	84.8 (56)	15.2 (10)	11.7 (66)	27.3 (3)	72.7 (8)	13.4 (11)
Sometime; Often; Everyday	48.2 (240)	51.8 (258)	88.3 (498)	33.8 (24)	66.2 (47)	86.6 (71)
Total	52.5 (296)	47.5 (268)	(564)	32.9 (27)	67.1 (55)	(82)
	*χ*^2^ = 209.76***			*χ*^2^ = 26.26**		
	**≤2 Years Since Diagnosis**			**≥3 Years Since Diagnosis**		
** After Cancer**	Never or Rarely	Sometime; Often; Everyday	Total	Never or Rarely	Sometime; Often; Everyday	Total
**Before Cancer**						
Never or Rarely	80.0 (28)	20.0 (6)	6.5 (17)	74.4 (32)	25.6 (11)	7.4 (43)
Sometime; Often; Everyday	43.5 (104)	56.5 (135)	93.5 (239)	48.8 (162)	51.2 (170)	92.6 (332)
Total	48.2 (132)	51.8 (142)	(274)	51.7 (194)	48.3 (181)	(375)
	*χ*^2^ = 83.03***			*χ*^2^ = 130.06***		
	**≤15 Years in Current Relationship**			**≥16 Years in Current Relationship**		
** After Cancer**	Never or Rarely	Sometime; Often; Everyday	Total	Never or Rarely	Sometime; Often; Everyday	Total
**Before Cancer**						
Never or Rarely	65.6 (21)	34.4 (11)	12.2 (32)	87.8 (36)	12.2 (5)	11.2 (41)
Sometime; Often; Everyday	43.5 (100)	56.5 (130)	87.8 (230)	48.6 (158)	51.4 (167)	88.8 (325)
Total	46.2 (121)	53.8 (141)	(262)	53.0 (194)	47.0 (172)	(366)
	*χ*^2^ = 69.77***			*χ*^2^ = 141.74***		

**p < .01; ***p < .001.

Note: Numbers in parentheses are the total number of participants in each category.

Participants with a reproductive cancer type were significantly more likely to report that sex occurred never or rarely after cancer (52.4%) compared to 32.5% of those with a non-reproductive cancer (χ^2^ (1, 648) = 11.42, *p* < .001), but no differences were found in these reports according age, years since diagnosis and years in current relationship.

#### Sexual satisfaction

Table 3 identifies the changes in sexual satisfaction after cancer, for women and for men, across reproductive and non-reproductive cancers, age band, years since diagnosis and current relationship duration. Both women (χ^2^ (1, 506) = 186.49, *p* < .001) and men (χ^2^ (1, 117) = 39.93, *p* < .001) rated their sexual relationship as significantly less satisfying after cancer, with 48.8% of women and 44.4% of men rating their current relationship as unsatisfying, compared to 6.7% of women and 4.3% of men before cancer. This finding was consistent across all age, cancer type, years since diagnosis and relationship length categories: ≤55 years of age (χ^2^ (1, 370) = 140.34, *p* < .001) and ≥56 years of age (χ^2^ (1, 250) = 84.15, *p* < .001); reproductive (χ^2^ (1, 541) = 217.36, *p* < .001) and non-reproductive cancers (χ^2^ (1, 76) = 10.32, *p* < .01); ≤2 years since diagnosis (χ^2^ (1, 262) = 91.46, *p* < .001) and ≥3 years since diagnosis (χ^2^ (1, 359) = 133.99, *p* < .001); and ≤15 years in current relationship (χ^2^ (1, 250) = 81.92, *p* < .001) and ≥16 years in current relationship (χ^2^ (1, 354) = 140.51, *p* < .001).

**Table 3:** **Reports of sexual satisfaction before and after the onset of cancer by gender, age, cancer classification, time since diagnosis and relationship duration**

**Sexual satisfaction rating**	**Women %**			**Men %**		
** After Cancer**	Highly Unsatisfying; Unsatisfying	Adequate; Satisfying; Highly Satisfying	Total	Highly Unsatisfying; Unsatisfying	Adequate; Satisfying; Highly Satisfying	Total
**Before Cancer**						
Highly Unsatisfying; Unsatisfying	58.8 (20)	41.2 (14)	6.7 (34)	40 (2)	60 (3)	4.3 (5)
Adequate; Satisfying; Highly Satisfying	48.1 (227)	51.9 (245)	93.3 (472)	44.6 (50)	55.4 (62)	95.7 (112)
Total	48.8 (247)	51.2 (259)	(506)	44.4 (52)	55.6 (65)	(117)
	*χ*^2^ = 186.49***			*χ*^2^ = 39.93***		
	**≤55 Years of Age**			**≥56 Years of Age**		
** After Cancer**	Highly Unsatisfying; Unsatisfying	Adequate; Satisfying; Highly Satisfying	Total	Highly Unsatisfying; Unsatisfying	Adequate; Satisfying; Highly Satisfying	Total
**Before Cancer**						
Highly Unsatisfying; Unsatisfying	50.00 (12)	50.0 (12)	6.5 (24)	66.7 (10)	33.3 (5)	6.0 (15)
Adequate; Satisfying; Highly Satisfying	50.6 (175)	49.4 (171)	93.5 (346)	42.6 (100)	57.4 (135)	94.0 (235)
Total	50.5 (187)	49.5 (183)	(370)	44.0 (110)	56.0 (140)	(250)
	*χ*^2^ = 140.34***			*χ*^2^ = 84.15***		
	**Reproductive Cancer**			**Non-Reproductive Cancer**		
** After Cancer**	Highly Unsatisfying; Unsatisfying	Adequate; Satisfying; Highly Satisfying	Total	Highly Unsatisfying; Unsatisfying	Adequate; Satisfying; Highly Satisfying	Total
**Before Cancer**						
Highly Unsatisfying; Unsatisfying	60.0 (18)	40.0 (12)	5.5 (30)	44.4 (4)	55.6 (5)	11.8 (9)
Adequate; Satisfying; Highly Satisfying	49.5 (253)	50.5 (258)	94.5 (511)	34.3 (23)	65.7 (44)	88.2 (67)
Total	50.1 (271)	49.9 (270)	(541)	35.5 (27)	64.5 (49)	76
	*χ*^2^ = 217.36***			*χ*^2^ = 10.32**		
	**≤2 Years Since Diagnosis**			**≥3 Years Since Diagnosis**		
** After Cancer**	Highly Unsatisfying; Unsatisfying	Adequate; Satisfying; Highly Satisfying	Total	Highly Unsatisfying; Unsatisfying	Adequate; Satisfying; Highly Satisfying	Total
**Before Cancer**						
Highly Unsatisfying; Unsatisfying	64.7 (11)	35.3 (6)	6.5 (17)	50.0 (11)	50.0 (11)	6.1 (22)
Adequate; Satisfying; Highly Satisfying	44.9 (110)	55.1 (135)	93.5 (245)	49.3 (166)	50.7 (171)	93.9 (337)
Total	46.2 (121)	53.8 (141)	(262)	49.3 (177)	50.7 (182)	(359)
	*χ*^2^ = 91.46***			*χ*^2^ = 133.99***		
	**≤15 Years in Current Relationship**			**≥16 Years in Current Relationship**		
** After Cancer**	Highly Unsatisfying; Unsatisfying	Adequate; Satisfying; Highly Satisfying	Total	Highly Unsatisfying; Unsatisfying	Adequate; Satisfying; Highly Satisfying	Total
**Before Cancer**						
Highly Unsatisfying; Unsatisfying	40.0 (8)	60.0 (12)	8.0 (20)	76.5 (13)	23.5 (4)	4.8 (17)
Adequate; Satisfying; Highly Satisfying	50.0 (115)	50.0 (115)	92.0 (230)	45.7 (154)	54.3 (183)	95.2 (337)
Total	49.2 (123)	50.8 (127)	(250)	47.2 (167)	52.8 (187)	(354)
	*χ*^2^ = 81.92***			*χ*^2^ = 140.51***		

**p < .01; ***p < .001.

Note: Numbers in parentheses are the total number of participants in each category.

No differences were found in the proportion of participants after cancer rating the sexual relationship as unsatisfying according to age, years since diagnosis and years in current relationship, although participants with a reproductive cancer type were significantly more likely to report unsatisfying sexual relationships after cancer (49.9%) compared to 35.9% of those with a non-reproductive cancer (χ^2^ (1, 621) = 5.36, *p* = .021).

#### Sexual activities

**Figure 1:**
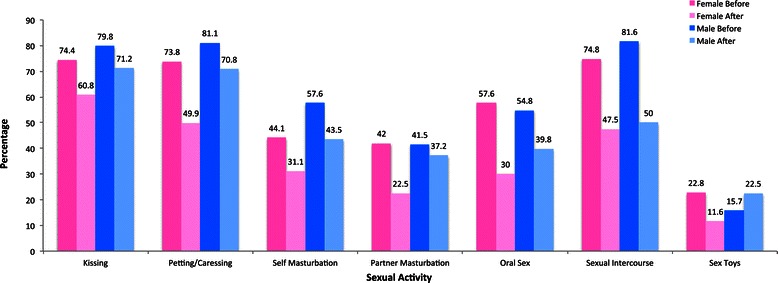
Changes in sexual activities before and after cancer by gender (%).

Seventy eight per cent of women and 76% of men indicated that their sexual activities had changed after cancer. The nature of changes in sexual activities is illustrated in Figure 1, combining reproductive and non-reproductive cancers.

A significant reduction in kissing (χ^2^ (1, 503) = 47.76, *p* < .001, 95% CI [.802,.290]), petting/caressing (χ^2^ (1, 493) = 100.65, *p* < .001, 95% CI [.032,.139]), self-masturbation (χ^2^ (1, 479) = 28.19, p < .001, 95% CI [.238,.536]), partner masturbation (χ^2^ (1, 479) = 64.61, *p* < .001, 95% CI [.170,.277]), oral sex (χ^2^ (1, 483) = 108.22, p < .001, 95% CI [.051,.165]), sexual intercourse (χ^2^ (1, 497) = 115.35, *p* < .001, 95% CI [.037,.138]), and sex toys (χ^2^ (1, 473) = 29.71, *p* < .001, 95% CI [.150,.442]) was reported by women. For men, a significant reduction in kissing (χ^2^ (1, 104) = 4.27, *p* = .039, 95% CI [.045,.926]), petting/caressing (χ^2^ (1, 106) = 4.76, *p* = .029, 95% CI [.090,.893]), self-masturbation (χ^2^ (1, 92) = 4.65, *p* = .031, 95% CI [.166,.924]), oral sex (χ^2^ (1, 93) = 7.04, *p* = .008, 95% CI [.077,.729]), and sexual intercourse (χ^2^ (1, 98) = 23.08, *p* < .001, 95% CI [.030,.320]) was reported. Although not statistically significant, the use of sex toys in men was the only sexual activity with a reported increase after cancer.

### Perceived causes of changes to sexual frequency and activities after cancer

**Figure 2:**
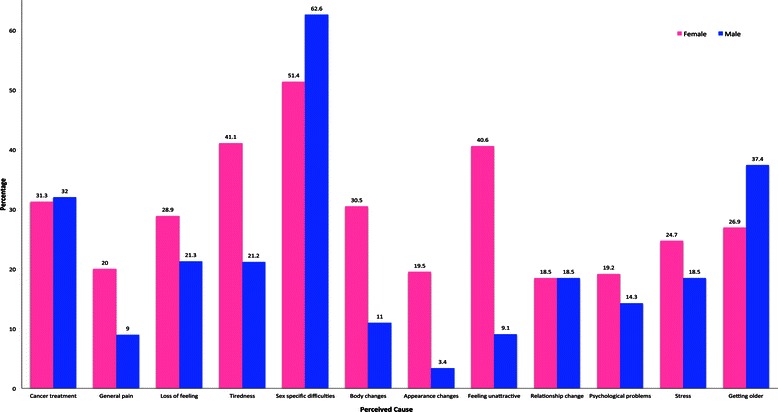
Perceived causes of changes in sexual frequency by gender (%).

Figure 2 contains responses to the closed ended question asking about perceived causes of reduced frequency of sexual activities, for women and men, combining reproductive and non-reproductive cancers. Sex specific difficulties (vaginal dryness and erectile dysfunction) were the most commonly reported explanation for both women and men, followed by tiredness and feeling unattractive for women, and surgery and getting older for men. Women were significantly more likely than men to indicate that general pain (*p* = .016; FET), tiredness (*p* < .001; FET), body changes (*p* < .001; FET), appearance changes (*p* < .001; FET), and feeling unattractive (*p* < .001; FET) were causes of changes to sexual frequency, whereas men were more likely to attribute change to getting older (*p* = .039; FET).

Four themes were identified in participant’s open ended survey and interview accounts of their perception of what had caused changes in sexual activities following cancer, summarised in Table 4. The most common theme, reported by approximately a third of the total survey sample, related to material changes to the body associated with sexual functioning, such as erectile performance, vaginal dryness or pain, the absence of sexual desire or arousal, or the aging process. A number of participants, approximately one eighth of the survey sample, also reported intrapsychic factors, such as stress, lack of confidence, low self-esteem, or fear, with a similar proportion identifying body image concerns, including feeling ‘hideous’ or ‘grotesque’, or worrying that a partner would find them unattractive. A number of participants, approximately one seventh of the survey sample, also identified relationship context as a factor that exacerbated difficulties, focusing on partner disinterest or rejection.

**Table 4:** **Perception of causes of changes to sexual activities after cancer**

Material changes to the body	I am still recovering from my operation 3 months ago. Also, I am still mildly incontinent and have erectile dysfunction (M, 54, hetero, prostate)
	Lack of interest on my part; tiredness; no feeling of arousal; no orgasm; vaginal dryness (W, 37, hetero, breast)
	Removal of clitoris due to a radical vulvectomy (W, 61, hetero, gynecologic)
	As a result of radiation treatment no erections, no fluid, plus even using Viagra. My penis is now quite small (M, 69, hetero, prostate)
	I didn’t really realize the radiation would affect my sexuality until it happened. I don’t think anyone can tell you what the pain discomfort and exhaustion will do to you (W, 61, hetero, digestive/gastrointestinal)
	A lack of testosterone and the natural aging process together, particularly the hormones, yeah, it does mean less sex (M, 77, hetero, prostate)
	We still hug each other and have a sort of intimacy. But –we have sex about once a year I would think, it’s barely a part of our relationship anymore. I mean, that may have something to do with getting older, I am 60 (M, 60, gay, prostate)
Intrapsychic factors	No erection. FEAR (M, 59, hetero, prostate)
	Confidence and self-esteem (W, 35, hetero, breast)
	I just don’t want to have her touch me because I don’t feel I deserve it or feel worthwhile (W, 45, lesbian, breast)
	I think there is a fear. I feel uncertain about sex (W, 50, hetero, gynecologic)
	Too stressed – I would rather sleep/read a book and be on my own for ‘me’ time. Now sex is a chore/duty (W, 44, hetero, skin)
	Prior to cancer and that we had good sex I think and a lot of digital pre-sex with lubricant and that, which did arouse her because otherwise she was slow to arouse… but of recent times, no. It was depression and lack of confidence and to a lesser extent, a lack of libido (M, 77, hetero, prostate).
	When I went through chemotherapy and a lot of the treatments I was a bit depressed as well, so that depression also turns you off wanting sex (W, 49, hetero, ovarian)
Body image concerns	Due to the lack of body parts I feel less like a sexual being and more like a breathing blob and that contributes to the fact that I have no inclination to have sex. (W, 48, hetero, gynecologic)
	My body is grotesque so I do not want anyone to see or touch me (W, 50, hetero, breast)
	I am hideous, my body is offensive and repulses me (W, 42, hetero, breast)
	I’m too embarrassed to let my partner put his fingers inside me in case the surgical scars/changes disgust him; I also worry that it will be uncomfortable (W, 30, hetero, gynecologic)
	you lose drive, you put on body fat, you lose muscle tone. Sexually you’re finished (M, 69, hetero, prostate)
Relationship context	
*Absence of partner support*	I just don’t look forward to it and would rather go without these days. Some if this has to do with my feelings for my partner. I don’t feel looked after or supported by him (W, 45, hetero, digestive/gastrointestinal)
	I find since my wife has been reluctant to provide physical support as and when I would like it, this in its self has put a great strain on our sexual relationship. (M, 57, hetero, prostate)
	I am stressed and therefore sex is the very last thing on my mind and the least thing I feel like - I would get more pleasure if my husband actually let me put my feet up - the last time I relaxed was when I was in bed for 3 days after my operation (W, 44, hetero, skin)
*Absence of partner sexual interest*	My partner won’t look at me or touch me (W, 46, hetero, breast)
	My husband has no interest in sex (W, 53, hetero, breast)
	My partner considered that following surgery our sex life was finished and she does not wish to resume (M, 73, hetero, prostate)

Key to abbreviations: gender (W = woman; M = man); age; sexual orientation (hetero = heterosexual, gay or lesbian); cancer type.

### Perceived consequences of changes to sexual activities after cancer

In response to an open ended survey and interview question asking ‘how have changes to your sexuality made you feel about yourself and your relationship?’, participants described intrapsychic consequences and changes to their intimate relationship.

#### Intrapsychic consequences of changes to sexuality

Approximately half of the survey participants identified intrapsychic consequences of changes to sexuality. These included disappointment at loss of sexual intimacy, frustration and anger, sadness, feelings of inadequacy and changes to sense of masculinity of femininity, as well as increased confidence and self-comfort, outlined in Table 5.

**Table 5:** **Emotional consequences of changes to sexuality after cancer**

Disappointment	I am disappointed in the loss of an almost perfect sexual relationship. (W, 68, hetero, breast)
	Disappointed. Feelings of loss and some resentment. Helplessness and hopelessness. (W, 58, hetero, breast)
	Disappointment that I am unable to enjoy and provide a sexual relationship as previously (W, 38, hetero, breast)
	Disappointed & frustrated. I went from being a healthy happy young woman to struggling with pain, unhappiness & fatigue most of the time with no-one interested anytime I express myself (W, 47, hetero, breast)
Frustration and anger	It’s horrible. I feel ripped off (W, 42, hetero, digestive/gastrointestinal)
	I feel like a failure and frustrated that this part of my life isn’t working like it used to. I’m angry that cancer has affected this too (W, 41, hetero, breast)
	I get annoyed that I can’t have sex anymore due to a large reduction in the size of my penis. (M, 62, hetero, prostate)
Sadness and depression	The loss of sexual function depresses me to the extent that life is meaningless and sometimes moves me toward suicide. If it was not for my wife and family and the effect my suicide would have on them I would have ended it long ago. (M, 71, hetero, prostate)
	The changes in our sex life have made me feel sad, not as sexy, and have caused us to be, I believe, less close. (W, 41, hetero, breast)
	I feel very sad, I miss the intimacy & closeness we use to have 12 years ago. My cancer 4 years ago has made the situation more difficult - my partner now sleeps in another bed & bedroom. I am heartbroken. (W, 51, hetero, breast)
Inadequacy	I feel inadequate – unable to express myself – and a whole heap of stuff that I am dealing with (M, 53, gay, prostate)
	Inadequate, physical horror with no breasts, angry, depressed (W, 71, hetero, breast)
	I feel like I have lost my femininity, first cancer mastectomy, ovaries removed, now loss of sexual desire (W, 35, hetero, breast)
	Inadequate, concerned that my partner thinks less of me, failing her, less complete (M, 59, hetero, prostate)
	Inadequate, incompetent, not worthy of the love and support of my wife, very sad, depressed that I cannot have sex with my wife, I feel useless to my wife. My wife would be better off without me. (M, 54, hetero, haematological/blood)
	My view of my masculinity has slumped (M, 65, hetero, prostate)
	Feeling less of a man (M, 65, hetero, prostate)
Feeling unattractive	My body looks and feels different and I have lost confidence in my body image. I don’t feel attractive and no longer have any desire to have a sexual relationship. (W, 50, hetero, breast)
	Cancer has impacted on how I view myself as a woman. Eg: hair loss is unattractive, Hickman line in place for months. My confidence in my appearance was lessened and I wondered how my boyfriend could possibly want to go near me. (W, 23, hetero, haematological/blood)
	I feel unsexy and self-conscious about my breasts. I worry that it will cause my partner to be disgusted by me. I’ve become a totally different person sexually and consider myself very unattractive. (W, 42, hetero, breast)
	I have gained weight so feel unattractive (W, 36, hetero, head and neck)
	Increased confidence in self. Appreciate life (W, 40, hetero, breast)
Increased confidence and self-comfort	I am more loving and more confident about life in general (W, 46, hetero, breast)
	My sexuality has changed for the better, I feel better about myself after cancer more accepting of my sexuality (W, 40, hetero, breast)
	I feel better about myself and my relationship since cancer (W, 41, hetero, breast)
	Freer. More alive. (W, 57, hetero, breast)
	I’m more comfortable with my body and self since the onset of cancer - I think because I’m on a big health kick and am in better shape than before - also less willing to let little things upset me, so if anything, our relationship is better. (W, 43, hetero, breast)

#### Relationship changes

A number of participants identified changes to their intimate relationship as a consequence of sexual changes experienced after cancer. These included relationship strain or termination, difficulties forming a new relationship, strengthened relationship, re-prioritisation of sex and re-negotiation of sexual intimacy, illustrated in Table 6.

**Table 6:** **Impact of changes to sexuality post-cancer on intimate relationships**

Relationship strain	I feel that if your partner lacks sensitivity and understanding to your feelings after a total hysterectomy then that relationship is not worth keeping. After the operation I felt no longer attractive as a woman. Intercourse became painful so I would avoid it. I found it easier to end the relationship, not having to worry about someone else’s feelings. (W, 62, hetero, gynecologic)
	My partner left me 6 weeks after mastectomy. He has huge fear of cancer- didn’t have the courage to face it, or support me. (W, 46, hetero, breast)
	Loss of libido or desire has created a wall. (M, hetero, 77, prostate)
	I feel as though I am constantly disappointing him. We are not as happy as we use to be. (W, 32, hetero, breast)
	The relationship is incomplete now, and I am not a satisfying partner anymore. I feel at risk of losing any relationship. (M, 58, gay, prostate)
	The relationship deteriorated rapidly. He expected me to be the same as before surgery and could not understand or didn’t want to what I was saying. He had an affair. (W, 62, hetero, genitourinary)
	It was such a new relationship when my cancer was diagnosed - we had been together only 3 months. I feel as though I am letting him down, being unable to have sex, and that this is too much for a partner to deal with at such an early stage of a relationship. I also feel that having this cancer “traps” him in the relationship - he may feel too guilty to leave even if he wanted to (M, 26, gay, haematological/blood)
Difficulty in forming new relationships	I don’t feel pretty enough or skinny enough for anyone who is willing to look my way. I haven’t had a relationship in all my life, but just recently stopped seeing this guy after a few weeks. I think I blame myself for not being attractive enough for keeping his attention. I feel like if I meet new guys and have a connection with them, then once they learn about my cancer it will probably turn them away. (W, 23, hetero, breast)
	My body was butchered … I have no relationship. (W, 51, hetero, breast)
	Made me uncertain and unwilling to enter a relationship (W, 61, hetero, breast)
	I feel ugly and that no one wants me and my husband left me when I was diagnosed with cancer haven’t found a new partner yet. (W, 42, hetero, breast)
	ED (erectile dysfunction) has greatly lowered my confidence in locating a new partner (M, 71, hetero, prostate)
	It makes me sad and a little depressed that I have no partner, more so since the cancer. It makes me feel like I will never have a partner again (W, 26, hetero, digestive/gastrointestinal)
Strengthened relationship	More open, prepared to deal with challenges by discussing with each other openly (W, 32, hetero, haematological/blood)
	I find life different – not being able to get an erection and maintain it. Pleasure is now derived by digital and oral sex for my wife. Emotionally our relationship has deepened. (M, 68, hetero, digestive/gastrointestinal)
	We are less emotionally and physically aroused but perhaps deeper in love and understanding (W, 75, hetero, breast)
	Even though I look at myself as deformed, he doesn’t and he shows that he loves me more often. Both in and out of the bedroom. (W, 32, hetero, breast)
	I’m very blessed to have a caring & loving husband who always tries to satisfy my needs. We both miss the spontaneity of our sex life since the onset of my cancer but we have a very strong and loving relationship which gets us through it all (W, 37, hetero, breast)
	My sexual partner has always wanted to have sex with me - this made me feel good about myself after my surgery and when my head shaved. He didn’t care - just loved my body anyway. He was great (W, 51, hetero, brain)
Re-prioritising sex	Sex is not a big deal anymore for either of us. We enjoy each other’s company regardless. (W, 60, hetero, gynecologic)
	I know my husband still loves me and cares for me. We are getting older and realize sex is not the most important thing in a relationship. We know we love each other no matter what happens. (W, 41, hetero, haematological/blood)
	Being sexuality active or not does not define me or my relationship. It’s just not that important to me any more (W, 42, hetero, breast)
	You’re naturally disappointed that you can’t sort of perform as you used to [chuckles], but you know it’s not a *major* problem with me sort of, as I say, getting on towards my 70s anyway (M, 68, hetero, myeloma)
	I went through menopause and had started to accept the fact that, that physical sex, penetration, all those things, were just not there as much as they had been, or nor did I want them as much as I had. It’s different when you’re [laughs] over 60 than when you’re 40 or 50 (W, 61, hetero, anal)
Renegotiating or redefining sex	I can’t get an erection but I think we have, I would rate it as nearly as good a sex life as before the operation. So you know, in terms of, it depends what, how hooked you are on penetration (M, 68, prostate)
	We were like, oh, two puppies playing together, even though I’m 59 and he’s 74. We sort of simulated sex - we’d get on top of each other and not actually have sex but, you know, sort of loving each other in a sex position (W, 59, hetero, lymphoma).
	You can’t reliably have normal intercourse. You’ve got to, you know sort of do other ways either, sort of, manually exciting or that sort of thing, oral sex and that type of stuff. So um … ah they’re the changes but we have been able to sort of work through that and so that it’s not a massive problem (M, 68, hetero, myeloma)
	In keeping the sexual relationship alive…that’s meant coming to terms with medication and cock rings and a whole range of sex aids that actually mean communicating pretty openly with, or very openly with my partner. (M, 65, gay, prostate)
	Well, I guess we sleep together, so that’s a good thing, and cuddle up, and touch, and that sort of thing is always good. (W, 59, hetero, ovarian)
	We hug a lot and we um, in bed, I would cuddle up as much as I can, [Pause] when he comes home, he always kisses me hello and so on. he’d just stop me in the kitchen and put his arms around me. I haven’t had an erection for nearly 12 weeks, and it’s impossible for me to get one. So that little aspect of intimacy is very important to me (M, 65, gay, prostate)

## Discussion

The aim of this study was to examine the subjective experience of changes to sexuality after cancer, as well as the perceived individual and relational causes and consequences of such changes, for women and men, across both reproductive and non-reproductive cancers and a range of relational contexts, using a mixed method design. The findings further develop the findings of previous research, which has primarily focused on either women or men with sexual and reproductive cancers, using quantitative survey methods.

Whilst reported levels of sexual dissatisfaction prior to cancer were comparable with Australian population norms [80], the majority of participants reported significant reductions in sexual satisfaction and frequency, as well as changes in a range of specific sexual activities, after cancer. Whilst a greater proportion of those with prostate, breast or gynaecological reported these sexual changes, the proportion of individuals reporting reductions in sexual frequency and satisfaction after cancer increased significantly for both the reproductive and non-reproductive cancers. This confirms previous research reports of cessation or reduction in sexual frequency after cancer, associated with reductions in sexual satisfaction [3,12,28,64,81], and refutes the notion that such changes are only a concern for individuals with cancer that directly affects the reproductive organs [43]. As there was no effect of time since diagnosis on reports of sexual changes, this confirms previous findings that sexual changes can be experienced at any stage of the cancer journey [16], and can be one of the most enduring negative consequences of cancer [82]. Concerns about sexual changes were also reported across age and gender, refuting the myth that older people are not sexual beings [83,84], and that sexuality is more of a concern to men than to women with cancer [85]. Whilst many older men and women reported distress in relation to sexual changes experienced following cancer, for some participants age was positioned as a *cause* of such changes, which allowed sexual changes to be positioned as normal or natural, resulting in a reprioritisation of sex within relationships. These findings suggest that broader cultural discourses which position older people as ‘naturally’ less sexual may serve a positive purpose for some individuals, allowing acceptance of sexual change after illness.

Whilst previous research on cancer and sexuality has focused on sexual intercourse [37,54], the present study adopted a broader definition of sex, examining both coital and non-coital sexual practices. Engagement in sexual intercourse was significantly reduced after cancer for both women and men, confirming previous research [13,18,86]. Engagement in non-coital sexual practices, including self and mutual masturbation, oral sex, and kissing, was also reduced, suggesting that once sexual intercourse becomes difficult or impossible, other forms of sexual intimacy may also cease. However, some forms of sexual intimacy did remain, even if reduced. Kissing and petting/caressing were reported to be the most common sexual activities after cancer for both women and men. When viewed in conjunction with a face-value increase in men’s reports of the use of sex toys, and qualitative accounts of the exploration of new sexual practices, or non-genital intimacy, this indicates sexual re-negotiation [87] or flexibility [88] following cancer on the part of some couples. This suggests that future research on sexuality and cancer should not only adopt a wider definition of sex to include non-coital sexual practices, but also examine strategies of sexual re-negotiation [55].

A substantial number of participants in the present study directly attributed changes in sexual frequency and sexual activities to the material consequences of cancer and cancer treatment, including vaginal dryness, erectile dysfunction, tiredness, loss of feeling, and general pain. However, participants also identified intrapsychic factors such as fear, stress, confidence and low self-esteem, as well as concerns about appearance, and relational factors, as causes of sexual changes. At the same time, a range of negative intrapsychic and relational factors were described as consequences of sexual changes experienced after cancer. This confirms previous reports that embodied sexual changes [78], psychological distress [6,45], relationship context [51,89,90], are associated with sexual difficulties after cancer, reinforcing the view that research on sexuality and cancer should adopt an integrative approach that acknowledges biological, psychological and relational factors [36,37,53]. The findings of the present study suggests that psycho-social and relational factors can be conceptualised as *both* causes and consequences of sexual changes experienced after cancer, which may operate in a vicious cycle of increasing difficulty and distress in the absence of information or support. This is in contrast to previous quantitative research which has examined psycho-social and relational factors as either predictors of sexual dysfunction after cancer e.g. [12,27,36,45], or as outcomes of sexual changes e.g. [56,91,92].

The material-discursive-intrapsychic model adopted in this research also acknowledges the discursive construction of gender and the sexual body in conceptualising the experience of sexual changes after cancer [78,93]. In this vein, many women participants attributed sexual changes after cancer to body image concerns, and to feeling unattractive as a consequence of sexual changes. This supports previous reports that cancer can serve as an ‘invisible assault to femininity’ [94], associated with diminished gender identity [59,95], and feelings of lack of sexual attractiveness and sexual confidence [96,97]. Socio-cultural constructions of idealised femininity normalise sexually attractive women as thin and young [98], with intact breasts signifying desirability [99]. Whilst such ‘emphasised femininity’ [100] is often unattainable, it is a core cultural ideal that shapes many women’s experiences of embodiment [101]. As the present study shows, these constructions impact on women’s sexual practices and subjectivity post-cancer – leading many of the participants feeling that they are now noncompliant with femininity, because they are reportedly ‘inadequate’, ‘fat’, ‘different’, ‘grotesque’ and ‘sexually unattractive’.

At the same time, a number of men also described concerns about masculinity, focusing on feelings of inadequacy, as reported in previous research on identity and sexuality in men with cancer [11,12,102,103]. Loss of erectile function can lead men to feel a change in their self-worth and manhood [7], with men who have prostate cancer reporting that they no longer live up to social expectations of masculine behaviour [104]. Men have also reported that their sexuality is ‘fractured’ post-cancer due to the onset of ‘failed’ sexual performance and diminished desire and pleasure [11]. In addition, men have reported feeling as though they are failing in their intimate relationship post-cancer, with erectile dysfunction seen as limiting the means by which they can ‘meet the needs of their partner’ [105,106]. Similar accounts were evident in the present study, in reports of perceived personal and relationship failure on the part of male participants- see also [93]. These accounts draw on discursive constructions of ‘real sex’ as coital penetration; described as the “coital imperative” [107,108] (pp44), (pp229), these socially constructed meanings serve to “set the horizon of the possible” in terms of sexual desire and behaviour [109], (pp16), provide the context within which individuals construct and experience changes to sexual feelings or behaviour following cancer.

Conversely, we found that a number of participants reported greater sexual confidence, a more positive self-image and increased relational closeness. Many of these participants reported being far less critical of their body post-cancer, less insecure about body image, as well as sexually empowered [78]. The responses of partners were a central part of this positive response, confirming previous research in this area [90,110,111], with many participants providing accounts that partner suggested acceptance of sexual changes, and continuation of partner interest and desire, was a helpful way of negotiating and dealing with the disruption of cancer. The importance of the discursive construction of the post-cancer sexual body was evident in accounts of relational negotiation of sexuality, wherein partners were reported to have positioned the changed sexual body as abject, or conversely, accepted sexual changes. These accounts draw on broader cultural discourse about sexual embodiment and illness, wherein breaches of bodily boundaries through leakage of fluid, surgical scarring, or the use of medical interventions such as a colostomy bag, signify disgust and decay, the anathema of sexual attractiveness or desire [112,113].

This study has a number of strengths and limitations. The strengths were the inclusion of men and women across a range of cancer types, ages, cancer stages and relationship contexts, to examine the subjective experience and consequences of sexual changes after cancer. The mixed method design, and relatively large sample for the qualitative component, is also a strength. Whilst previous research has focused on the heterosexual population, the present study included individuals who identified as gay, lesbian and polysexual, confirming that sexual changes also affect this hitherto “hidden population” [114]. However, as sample size of LGB individuals precluded sub-group analysis, further research is needed to examine the causes and consequences of sexual changes and renegotiation after cancer within a larger population of gay, lesbian, bisexual, and transgender individuals, who have been described as an “overlooked health disparity” ([115], p1009) in the context of cancer. Further limitations were the use of a single item to measure sexual satisfaction, and non-standardised measures of changes to sexual frequency and sexual activities. The retrospective nature of data collection, asking participants to report on perceived changes pre-post cancer, was also a limitation; prospective analysis of sexual changes through the course of diagnosis and treatment would overcome this. The over representation of women with breast cancer, and low representation of men in the sample is also a limitation. Whilst breast cancer is the most common cancer affecting women, there is an under-representation of prostate cancer, the most common cancer affecting men, as well as other common cancer types [1], including respiratory, skin, gastro-intestinal and head and neck cancers. This may be because individuals with non-reproductive cancers are less likely to volunteer for a study on sexual changes, as well as effective strategies of participant recruitment on the part of breast cancer organisations. Future research which specifically targets non-reproductive cancers is needed to examine the subjective experience of sexual changes after cancer, and examine whether the present findings can be generalised.

### Clinical implications

The findings of the present study have a number of clinical implications. Firstly, they confirm the importance of health professionals and support workers acknowledging sexual changes when providing health information and developing supportive interventions. There is evidence that information about sexual changes and strategies of re-negotiation is often not forthcoming in clinical consultations [44,116], in particular for women and for individuals with a non-reproductive cancer [43], and as a result patient sexual concerns are unaddressed. Equally, whilst a range of one-to-one and couple interventions have been developed to address sexual difficulties after cancer [41,117-120], these are primarily focused on the functioning of the body for individuals with breast, prostate or gynaecological cancer. There needs to be an expansion of such support into non-reproductive cancers, to address the concerns and support needs of patients and their partners, across cancer stage, age, and relationship status, with the impact of cancer on sexuality acknowledged by researchers and health professionals working across the whole spectrum of cancer care.

Secondly, our findings suggest that psychological interventions aimed at reducing distress and improving quality of life after cancer should include a component on sexual well-being, and conversely, that sexual interventions should incorporate components on psychological and relational functioning. Clinicians need to acknowledge the complex meanings individuals with cancer attribute to sexual changes, in the context of their individual lives and relationships, rather than solely focusing on the functioning of the sexual body [52]. The implication of the finding that partners play a key role in perception of changes to sexuality points to the need for health professionals to recognise the relational and intersubjective nature of sexuality so that discussion of sexuality between people with cancer and their partners is normalised and legitimated [121]. As previous research has reported that couple therapies which facilitate relational coping and communication in the context of cancer are effective in reducing distress e.g. [122-124], couple focused information and supportive interventions to reduce sexual concerns may be beneficial for those in a sexual relationship. However, sexual concerns also affect single people, as was evidenced by accounts of difficulties in forming new relationships in the present study. Information and support services also need to acknowledge and address the specific needs and concerns of single people, a group whose sexual concerns are often overlooked by clinicians [43,125].

Thirdly, our findings suggest that clinicians should adopt a broad conceptualisation of sex when discussing sexual changes, and sexual renegotiation after cancer, rather than focusing on coital sex. There have been previous reports of renegotiation of sexual practices following cancer, with men with prostate cancer reporting “different” and “better” sex after treatment ([126], p323), expressing intimacy through oral sex and touch, rather than penetration [127,128]. Research with partners of people with cancer has also reported renegotiation of sex to include practices previously positioned as secondary to “real sex”, such as mutual masturbation and oral sex [42,110]. This suggests that couples can resist the “coital imperative” ([108], p.229), the biomedical model of sex enshrined in definitions of ‘sexual dysfunction’, which conceptualises sex as penis-vagina intercourse. However, little attention has been given to renegotiation of sexual practice or intimacy after cancer, which has led to a plea for more attention to be paid to “successful strategies used by couples to maintain sexual intimacy” ([129], p142).

## Conclusions

Whilst previous research on cancer and sexuality has primarily focused on women with breast or gynaecological cancers, or men with prostate or testicular cancer, the findings of the present study demonstrate that post-cancer sexual and body image issues are also a concern for many women and men with non-reproductive cancers – including those in this sample with haematological, gastrointestinal, neurological, skin, respiratory, and head and neck cancers, across a range of age-groups, cancer stages and relationship contexts. For many individuals, these changes were a source of distress or relationship difficulty. This suggests that acknowledgment of sexuality should be on the agenda of cancer researchers, clinicians and policy makers, in order to facilitate prevention and intervention strategies that aim to reduce distress associated with sexual changes experienced after cancer and assist with sexual renegotiation.
